# Miniaturized Parasitic Loaded High-Isolation MIMO Antenna for 5G Applications

**DOI:** 10.3390/s22197283

**Published:** 2022-09-26

**Authors:** Kiran Chand Ravi, Jayendra Kumar

**Affiliations:** School of Electronics Engineering, VIT-AP University, Amaravati 522 237, Andhra Pradesh, India

**Keywords:** 5G antenna, microstrip antenna, mutual coupling, MIMO antenna, parasitic loaded antenna

## Abstract

In this paper, a multiple-input–multiple-output (MIMO) antenna is reported for 5G frequency range-2 (FR-2), 28 GHz bands. The MIMO antenna is developed in multiple iterations, including single-element design, cross-polarization reduction, and mutual coupling reduction. Initially, a single-element coplanar edge feed rectangular patch antenna is designed and the E-plane cross-polarization is reduced by −13 dB by trimming the forward corners of the patch. The ground plane is truncated to improve the −3 dB half-power-beamwidth (HPBW). A multi-wavelength spiral inspired parasitic surrounding the single element antenna is loaded, and performance analysis is performed. This parasitic element is used for self-field cancelation for the MIMO configuration. Two MIMO configurations, one with linear and the second with inverted elements, are developed and investigated. The first configuration is found to have better isolation of less than −25 dB compared to the −20 dB of the second configuration. Similarly, the gain of 4.8 dBi, the bandwidth of 3 GHz, envelope correlation coefficient (*ECC*) of 0.01, and diversity gain (*DG*) of 9.99 dB are superior to the second configuration. To validate the work, one of two MIMO configurations is fabricated and good agreement is found between simulation and measurement results.

## 1. Introduction

The global implementation of Fifth-Generation (5G) communication systems is beneficial for telecom businesses, but it will be fraught with difficulties. The proliferation of linked devices and the rise of the Internet of Things (IoT) will probably lead to a tremendous increase in data from mobile locations. New applications such as Industrial Internet of Things (IIOT), network-based autos and real-time machine-to-machine communication will demand higher data ratings. A lower-latency rate will permit connected devices to be used in critical applications such as healthcare and smart utilities, as the reaction time of 5G systems are estimated to be less than one millisecond [1]. The frequency range-1 (FR-1), 3.5 GHz frequency bands are being used for area-wide services. The frequency range-2 (FR-2), 26–28 GHz frequency bands must be used for mobile services to provide much a higher data rate: nearly 10 times that of the existing Fourth-Generation (4G) technology. During the evolution of 4G communication systems, no major technological shifts or discontinuities were required.

Antenna technology evolved from an exterior antenna to an interior antenna, necessitating changes to the antenna. Various applications have been covered through multiple single-band or dual-band antennas or through the diversity of multiple-input–multiple-output (MIMO) systems. For some applications, 5G implementations, on the other hand, allow for a tenfold increase in frequency [2]. This is a big shift from prior technologies, and it will present both obstacles and opportunities. The operating wavelength is much smaller, resulting in smaller individual array elements; thus, beam formation and beam steering using antenna arrays will be conceivable. The wavelength (λ) at these high frequencies is less than 10 mm; hence, the device is a multi-lambda platform [3]. As a result, antenna placement becomes considerably more important, replacing the integration component that was important in previous technologies. Because of the more complicated components of 5G development, it is also necessary to test different implementations to detect and change trade offs as efficiently as possible [4]. The fabrication of these millimetre-wave antennas becomes challenging due to wavelength-comparable size, and the thin metal traces will also be affected by climatic conditions.

Various approaches and methods have been proposed in the literature, such as electrically thick substrates, probe-fed antennas, and slotted antennas [5,6], with the aim of realizing half- or multi-wavelength antennas. In [7], 28 GHz compact phased array antenna has been discussed. Gain improvement can be obtained by using an array technique [8], but design complexity is the critical issue. The massive Multi-Input and Multi-Output (MIMO) proposed in [9] is promising 5G technology that has been intensively explored to improve data transmission speed and resistance to multiple pathways fading. The transmitter or receiver in a MIMO system must have two or more antenna elements. A tilted combined beam antenna for 5G applications is discussed in [10]. Multiple antennas, on the other hand, have drawbacks in that they increase the size of the system and deteriorate the isolation between them, resulting in a distorted radiation pattern and a reduction in channel capacity [11]. All relevant parameters must be considered and maintained when designing a MIMO antenna. It is most challenging to design a high-gain antenna at higher frequencies. A Multiband MIMO Microwave and millimeter-wave antenna system employing a dual-function tapered slot structure is reported in [12]. The FR-2 band 5G communication is still in development and planning stages. For successful implementation, huge efforts and research is required on various elements of 5G including antennas. Different low-frequency patch antenna performance enhancement techniques, such as partial ground plane [13], cross-polarization techniques [14], and gain and bandwidth enhancement [15,16,17] can be implemented in the FR-2 band to develop efficient antennas for 5G applications.

In this work, a novel mutual coupling field cancellation MIMO antenna structure is developed for the 28 GHz, FR-2 5G band. The cross-polarization of the single-element rectangular patch antenna is significantly reduced by precisely trimming the forward corners of the radiating patch. To improve the HPBW of the single element, the ground plane is truncated. The truncation of the ground plane reduces the gain and the bandwidth of the single element. However, when the parasitic is loaded in a later stage, the gain of the antenna is resituated. Now, two-element MIMO antennas are developed by placing these low cross-polarization single-antenna elements in different positions on single chases. A comparative analysis of the linear two-element antenna and the inverted arrangement of single elements is presented. The proposed field cancelation technique is successful, and antennas has a acceptable realized gain, good co- and cross-polarization, and isolation between antenna elements. The bandwidth of the antenna is reasonably high, allowing it to cover all FR-2 band 5G applications.

The paper is organized as follows: following the introduction, the design flow and parametric analysis of a single-element antenna and its performance evaluation of different iterations are presented in Section 2.1. Different MIMO configurations, their parametric analysis, performance evaluation, and comparative results are presented in Section 2.2. The prototype and comparative simulated and measurement antenna performance parameters are discussed in Section 3. Preceding Section 3, the work is concluded in Section 4.

## 2. Antenna Design

**Figure 1:**
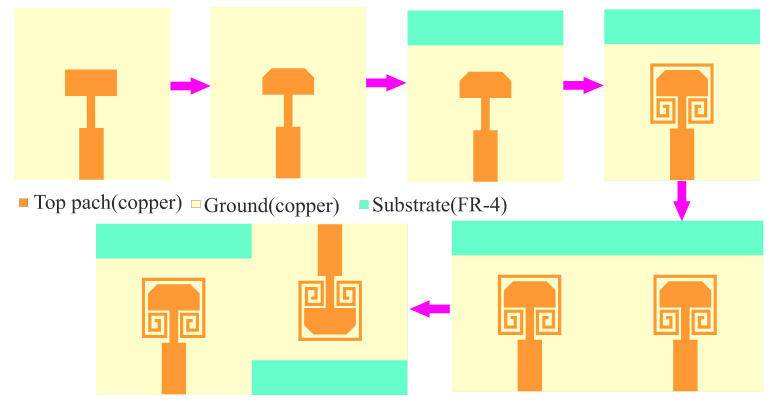
The design flow of the proposed work.

In this Section, the design and analysis of single elements, as well as MIMO configurations, are discussed. The effect of design parameters is investigated and the results are observed. Discussions are presented regarding the design parameters and their effect on the antenna performance. The proposed design is derived in five different iterations, as shown in Figure 1. The reference design of the proposed work is a Co-planar Waveguide (CPW) feed rectangular patch antenna. The CPW consists of a 50 Ω transmission line and another quarter-wavelength impedance transformer. The reference antenna has a dual-band resonance but overlapped co- and cross-polarization isolations. To improve the isolation between co and cross-polarization, the forward corners of the rectangular patch are trimmed. The trimming of the edges has also improved the antenna bandwidth, but reduced the antenna gain. Further, the ground plane of the trimmed patch antenna is defected, improving the gain, and the single element has sufficiently good bandwidth, co- and cross-polarization isolation, and gain. Later, two-element antennas are developed and investigated for better performance. The design and analysis of single-element and MIMO antennas are discussed in the following Sections.

### 2.1. Spiral-Inspired Parasitic Loaded Single Element Antenna

**Figure 2:**
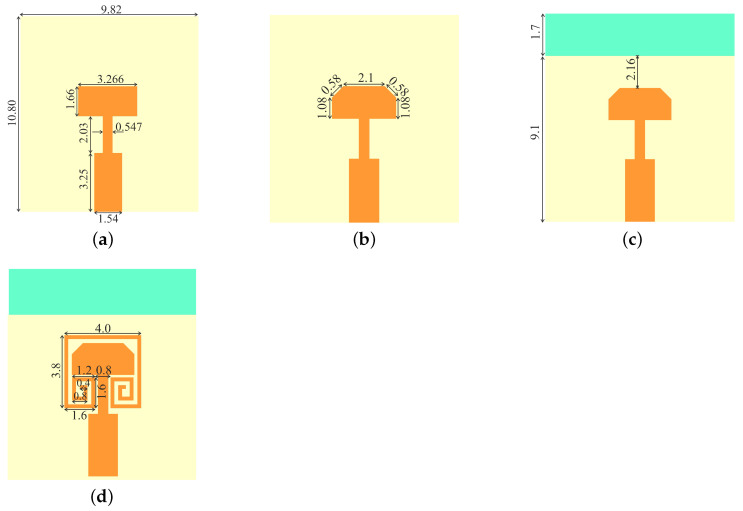
Layout of the antenna configurations (Units mm) (**a**) Reference model (**b**) Intermediate model (**c**) Intermediate model with partial ground (**d**) With parasitic patch.

The single-element antenna is designed in four iterations, as shown in Figure 1. The layout of all four iterations is shown in Figure 2. As a part of the investigation, the design flow starts with the primary model fed by a 50 Ω microstrip line. Firstly, we design and demonstrate the reference model(primary antenna) resonating at 28 GHz, and FR-4 epoxy material with a thickness of 1.6 mm is utilized as a substrate.

**Figure 3:**
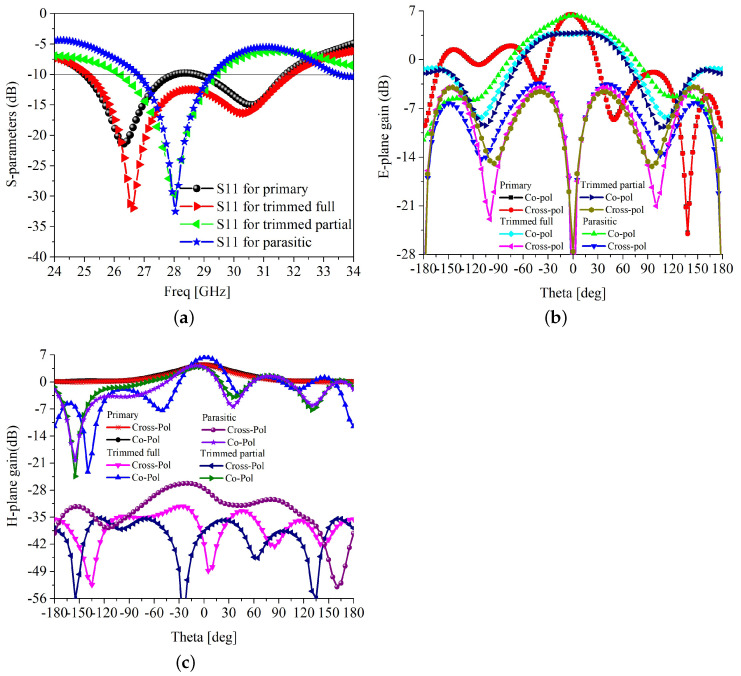
Antenna characteristics of different iterations (**a**) *S*_11_ (**b**) E-plane (**c**) H-plane.

The forward corners of the radiating rectangular patch of the primary antenna have been trimmed, and that generates a new model (trimmed model) to suppress the cross-polarization. The next iteration includes truncation of the ground plane to enhance the HPBW, resulting in a new model, trimmed partial ground. A multi-wavelength spiral parasitic patch has been inserted around the top patch, which leads to the final single-element model in the fourth iteration. The design attributes of these four iterations are the length and width of the patch, the length of the cut in the patch corner, the length of the ground, and the spiral-inspired parasitic design parameters, *a*, *b*, *c*, and *d*, respectively. Using these design attributes, the operating frequency, cross-polarization level, HPBW, and isolation between MIMO elements can be controlled. The simulated results for the four models are generated using the High-Frequency Structural Simulator Software (HFSS) tool. The scattering parameter *S*_11_ of the single element in all four iterations is shown in Figure 3. The primary design and the trimmed version antennas have a much wider bandwidth, covering from 25 to 31.6 GHz, as shown in Figure 3a. Meanwhile, the single element with the truncated ground and parasitic loaded designs has a narrower bandwidth, covering from 26.7 to 29.2 and 26.2 to 29.5 GHz, respectively. In an ideal rectangular patch antenna, the radiation occurs only from the width of the patch, and the radiation from the length is null. However, in a practical antenna, some radiation occurs from the longitudinal side, which is stronger at the forward corners of the patch. Thus, by trimming these two corners, the cross-polarization level can be significantly reduced. The E-plane side-lobe level (SLL) is also improved by trimming the forward edges, as shown in Figure 3c. The H-plane SLL is higher, which needs to be improved for better performance.The electric and magnetic field (E and H-field) broadside radiation pattern at 28 GHz of the different iterations of the single-element antenna is shown in Figure 3b,c. It is clear in Figure 3b that after trimming the forward corners of the rectangular patch, the E-plane cross-polarization level is reduced to −10 dB in the −3 dB HPBW range. The H-plane radiation experiences higher isolation of above 30 dB after trimming the corners. However, the transverse magnetic (TM) mode is dominant in a rectangular patch antenna; thus, co-and cross-polarization isolation is always better in H-plane than E-plane. Additionally, the trimming of the corners degrades the antenna gain but improves the −3 dB HPBW in E-plane. The gain is further improved by partial ground in the next iteration. In the H-plane, the HPBW is reduced due to the trimming of the corners, which is less likely. Another adverse effect of the trimming is that the bandwidth is reduced from 6.6 to 2.2 GHz, as shown in Figure 3a. However, the bandwidth of 2.2 GHz is large enough to cover many applications in FR-2 5G bands. These low cross-polarization and wider HPBW single elements have to be used to develop MIMO antennas. Thus, a spiral-inspired parasitic element is placed around the trimmed rectangular patch, as shown in Figure 2d, for mutual field cancelation. In the following sections, two single-element antennas are placed on common substrates to realize two-element MIMO antennas. The detailed analysis is presented in Section 2.2.

**Figure 4:**
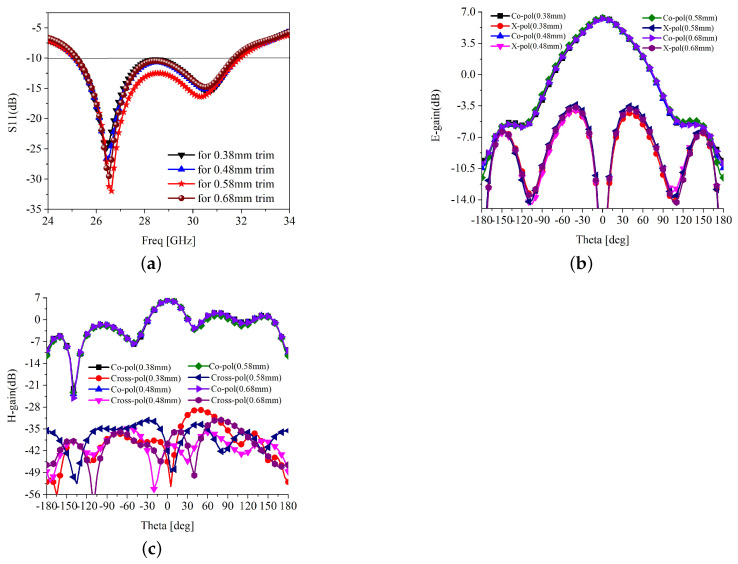
Impedance matching and E and H-plane radiation at various level of trimming (**a**) *S*_11_ (**b**) E-plane radiation (**c**) H-plane radiation.

**Figure 5:**
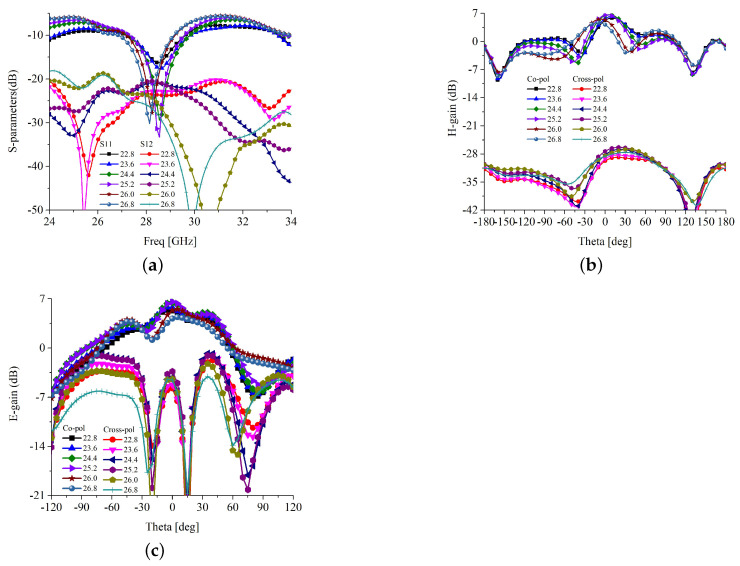
Impedance matching and E and H-plane radiation for different length of parasitic (Lspiral). (**a**) *S*_11_; (**b**) H-plane radiation; (**c**) E-plane radiation.

The effect of the attributes, trimming level, and integration of spiral-inspired parasitics are shown in Figure 4 and Figure 5. It is clear in Figure 4a that the trimming level has some effect on the impedance matching. It is observed that for the trimming of 0.58 mm at 45${}^{{}^{\circ}}$, the antenna has the best *S*_11_ curve, resulting in the best matching. Thus, the trimming level is fixed to 0.58 mm. The trimming level has no significant effect on the E-plane radiation, but affects the H-plane cross-polarization radiation, as depicted in Figure 4b,c. However, the co- and cross-polarization isolation is more than 30 dB in all cases; thus, the effect of trimming level can be ignored. The scattering parameters against the different length of the parasitic element are shown in Figure 5a. It is understood that the length of the parasitic needs to be carefully optimized to obtain better impedance matching, bandwidth, and isolation. For a length shorter than 2.2λ, the impedance matching, bandwidth, and isolation are poor. Beyond 2.2λ lengths, the performance of the antenna is significantly improved. However, the parasitic length beyond the 2.2λ deviates the main lobe of the H-plane radiation, as shown in Figure 5b. Meanwhile, the E-field radiation and H-plane cross-polarization have a negligible effect on parasitic length, as presented in Figure 5b,c, respectively. Similarly, other design attributes of the proposed antenna are optimized to obtain the best possible impedance matching, bandwidth, gain, and co- and cross-polarization isolation.

### 2.2. Design of MIMO Antenna

**Figure 6:**
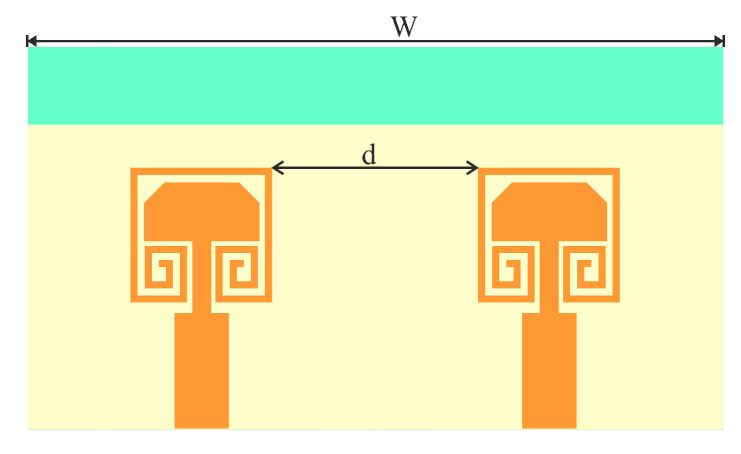
MIMO configuration-I Layout.

**Figure 7:**
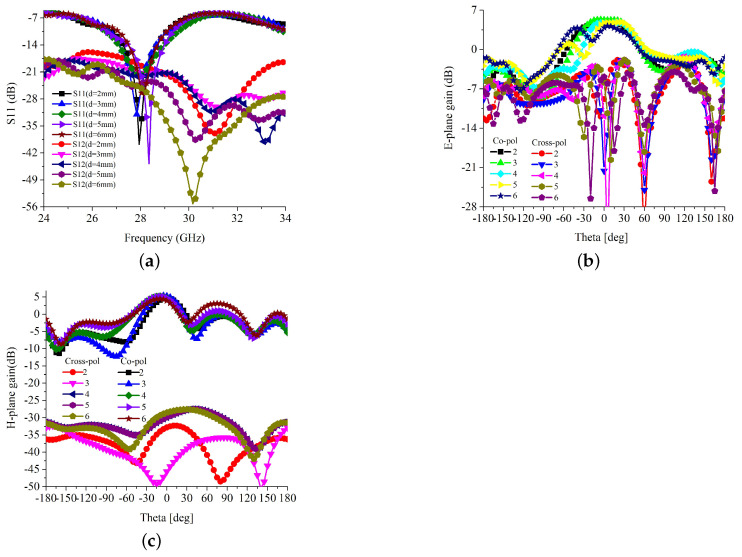
Radiation pattern of the MIMO configuration-I against separation (*d*) between antenna elements (**a**) *S*_11_ and *S*_12_; (**b**) E-plane; (**c**) H-plane.

The two-element MIMO antenna is investigated in two different configurations. One is a traditional linear arrangement, and the other is an inverted arrangement of the single elements. The layout of linearly arranged MIMO antenna is shown in Figure 6. Two single-element antennas are placed at a separation *d* on a common substrate of width *w*. The mutual coupling analysis is performed and presented in Figure 7a–c. The scattering parameters against the separation between antennas are presented in Figure 7a. It is clear in Figure 7a that the impedance matching and the operating frequency of the antenna are slightly deviated against the separation between antennas. The severe effect of the antenna separation can be seen on *S*_12_, with mutual coupling. As the separation between antennas is increased, the mutual coupling, *S*_12_, significantly reduces. Thus, a trade-off between the size of the antenna and isolation between antenna elements has been established [18]. For better isolation, the separation between antenna is fixed to $0.54\lambda_{0}$, and the substrate width is fixed to 19.82 mm. The overall size is sufficiently small to fit in consumer electronics devices. The antenna performance against the separation at 28 GHz is also presented in Table 1. It is clear from Table 1 that the antenna has considerable impedance matching, gain, bandwidth, and isolation for separation higher than 5 mm.

The E-field co- and cross-polarization isolation against the antenna separation is depicted in Figure 7b. It is observed that the separation between antennas has not had a significant effect on the E-plane co- and cross-polarization radiation. The H-field co and cross-polarization isolation against the antenna separation are depicted in Figure 7c. Likewise, the co-polarization radiation of MIMO configuration-1 has a trivial effect on the antenna separation. The H-plane cross-polarization is notably affected by the antenna separation. For the smaller separation, MIMO configuration-1 has a lower cross-polarization level due to the cross-polarized fields’ mutual cancelation. However, due to the solid mutual coupling in the co-polarization, the antenna elements cannot be placed within the near field regions.

**Figure 8:**
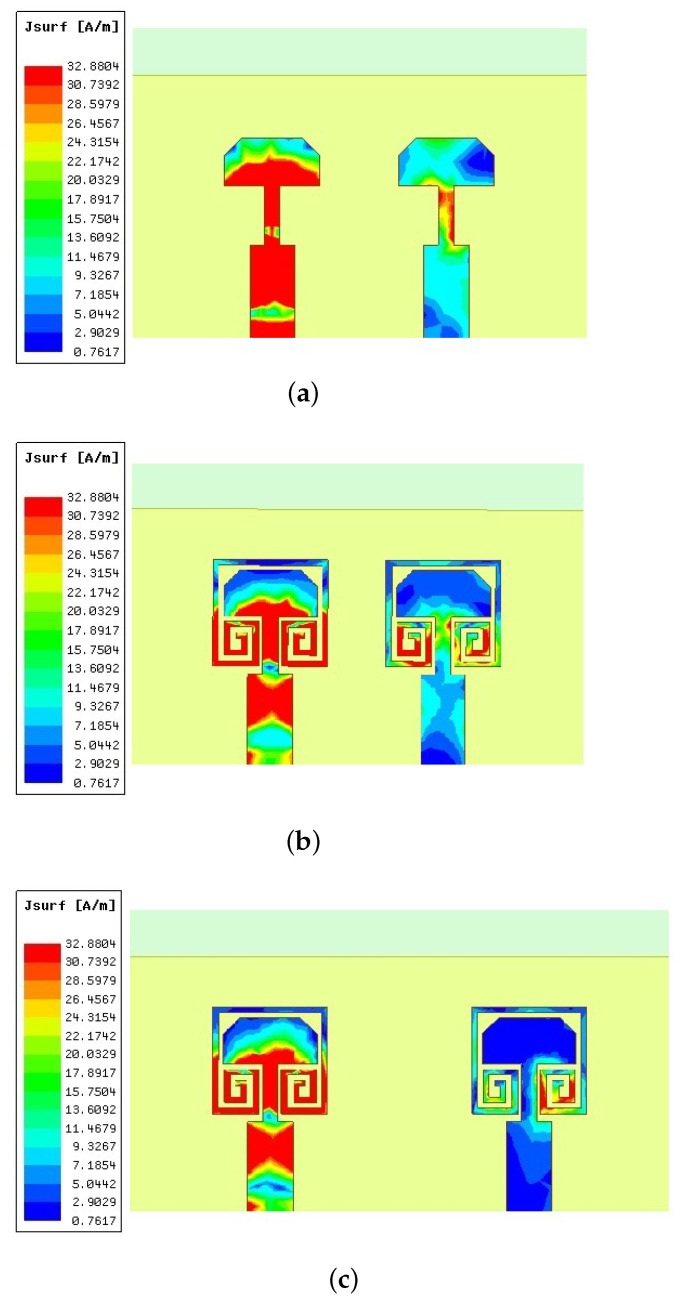
Surface current distribution of different configurations at 28 GHz. (**a**) Without parasitic ($d=0.19\lambda_{0}$) (**b**) With parasitic ($d=0.19\lambda_{0}$) (**c**) With parasitic ($d=0.54\lambda_{0}$).

The surface current distribution with and without parasitic loading and against the separation between antenna elements are shown in Figure 8. It is clear in Figure 8a that the second element (right side) has strong mutual excitation. Strong surface current distribution can be observed on the feed line, as well as the trimmed radiator. This is due to the antenna elements being placed just 2 mm apart, lying within the near-field range at 28 GHz. When the spiral-inspired parasitic is loaded, the current distributions on the feed line and radiating patch have been significantly reduced. This confirms the success of the proposed field cancelation technique. These spiral-inspired parasitic are $180^{{}^{\circ}}$ out of phase with the antenna elements, thus canceling the mutually exciting fields, resulting in improved isolation and MIMO performance. To further improve the isolation, the separation between antenna elements is slightly increased beyond the near field region (≈>λ/2), 5.8 mm. As the antennas are shielded by external parasitic elements, as well as being placed in far-field regions, the mutually exciting current is very poor, as shown in Figure 8c. The MIMO performance parameters, envelope correlation coefficient (*ECC*), and the diversity gain (*DG*) are calculated using the scattering parameters of the antennas, as expressed in Equations (1) and (2).

(1) $$\begin{array}{c} ECC=\frac{|S_{11}^{*}\times S_{12}+S_{21}^{*}\times S_{22}{|}^{2}}{\left(1-\right|S_{11}{|}^{2}-|S_{21}{|}^{2})(1-|S_{22}{|}^{2}-|S_{12}{|}^{2})} \end{array}$$

(2) $$\begin{array}{c} DG=10\sqrt{1-{\left(ECC\right)}^{2}} \end{array}$$

**Figure 9:**
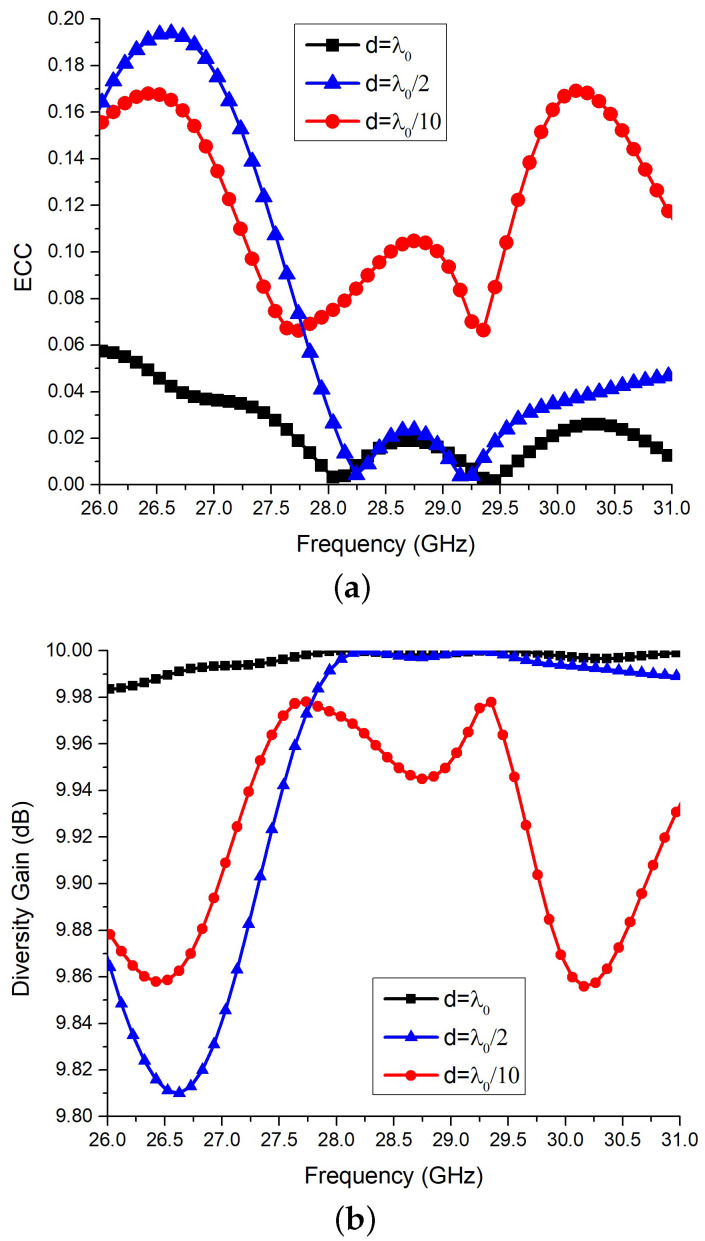
Simulated performance characteristics of MIMO-1. (**a**) *ECC* (**b**) *DG*.

The *ECC* and *DG* of the MIMO configuration-1 against the antenna separation are shown in Figure 9a,b. It is clear in Figure 9a,b that the larger the separation between antenna elements, better the MIMO performance parameters. For the largest separation of $2\lambda_{0}$, the MIMO configuration-1 has nearly zero *ECC* and *DG* around 10 dB, as shown in Figure 9.

**Figure 10:**
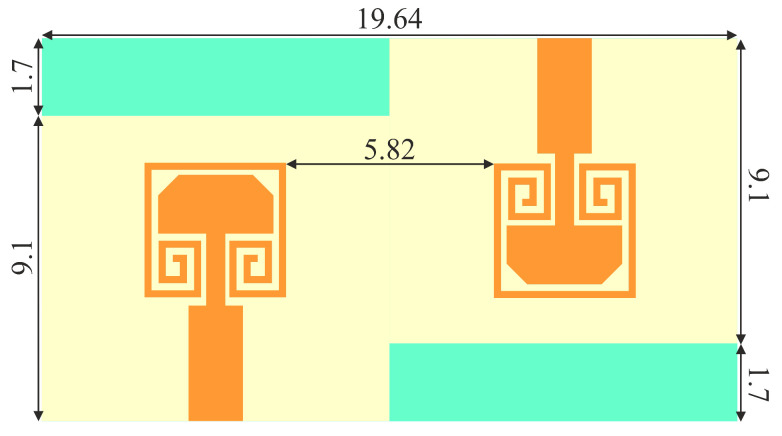
The layout of the inverted antenna element MIMO-2 configuration.

**Figure 11:**
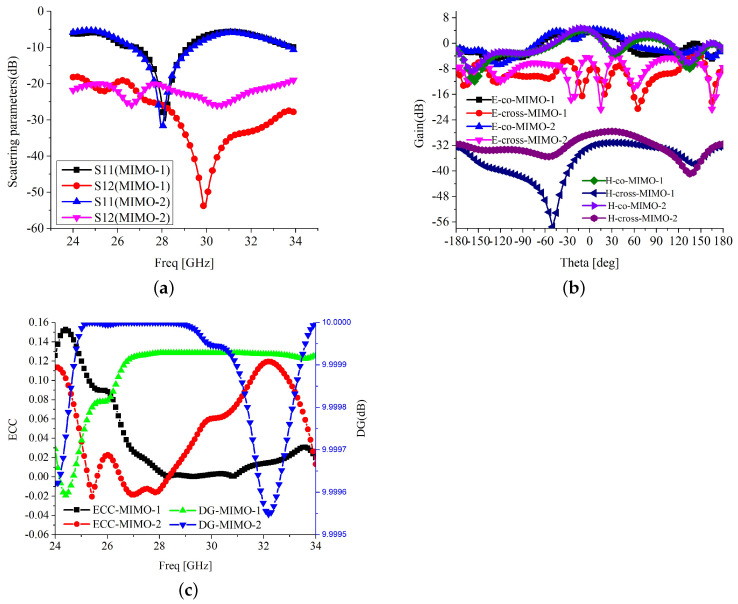
Comparative results of MIMO 1 and 2 configurations (**a**) S-parameters (**b**) Radiation pattern (**c**) MIMO performance parameters.

Further, another two-element MIMO configuration is developed, in which the antenna element fields are $180^{{}^{\circ}}$ out of phase, as depicted in Figure 10. The antenna elements are placed in inverted form and separated at a distance of $0.54\lambda_{0}$, as in MIMO configuration-1. As the antenna elements are out of phase, the spiral-inspired parasitic of the second element comes into phase with the first antenna. Thus, it generates the mutually exciting current in the second element and reduces the isolation between the antenna elements. This confirms the active role of the spiral-inspired parasitic in mutual coupling control. However, the antenna elements are placed in far-field regions so the isolation still remains in the acceptable range. The comparative scattering parameters, e and H-field radiation fields, ECE, and *DG* of the MIMO configurations 1 and 2 are shown in Figure 11.

Figure 11a shows that the MIMO configuration 1 has better isolation above 25 dB due to the out-of-phase characteristics of the spiral-inspired parasitic. The parasitic elements are mutually excited by the radiating field of the patch. In a patch antenna, the current flows through the length and radiates from the width. Thus, the parasitics are excited along the width and the current flows opposite the patch current. The terminating sides of the parasitic have stronger radiating fields opposite to the fields caused by a mismatch between the feed line and the patch. Thus, these parasitics are canceling the standing waves, which is a major source of mutual coupling. The MIMO configuration 2 isolation is significantly reduced due to the parasitic in-phase response. The impedance matching and bandwidth are slightly deviated in either configurations. Due to the higher mutual coupling, the gain of the MIMO configuration 2, 4.1 dBi is poorer than configuration 1, 4.8 dBi, as presented in Table 2.

**Figure 12:**
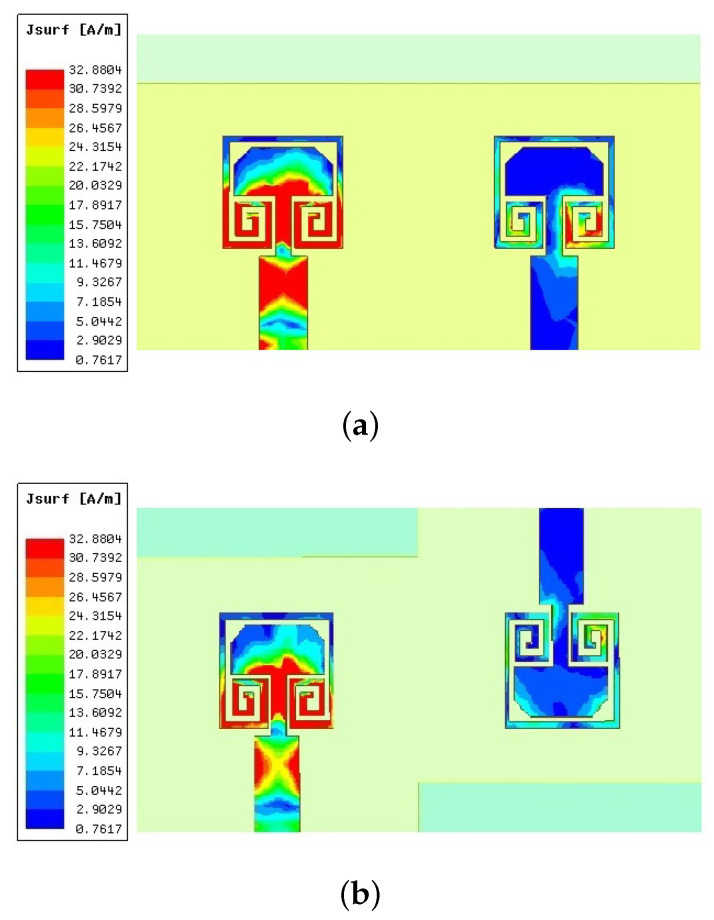
Surface current distribution of MIMO-1 and 2 configurations (**a**) MIMO-1 (**b**) MIMO-2.

The E and H-field radiation pattern of both configurations are shown in Figure 11b. The E and H-field co-polarization and E-field cross-polarization are not affected by the inversion of one antenna element. The H-plane cross-polarization is reduced in the case of MIMO configuration 2 due to the out-of-phase H-field cross-polarization radiations. The *ECC* and *DG* of both configurations are shown in Figure 11c. The *ECC* of configuration 1 is greatly superior to configuration 2. To visualize the mutually exciting current on both configurations, the surface current distribution is drawn and shown in Figure 12. It is clearly visible that the out-of-phase antenna element arrangement (configuration 2) has a stronger mutual surface current at the trimmed corners compared to configuration 1. For configuration 1, the strong surface current is on the parasitic rather than the antenna element. For the second configuration, the strong surface current is on the antenna element and the parasitic element has lower surface current distribution. This confirms the out-of-phase parasitic coupling theory and ensures the successful operation of the proposed design.

## 3. Experimental Results

**Figure 13:**
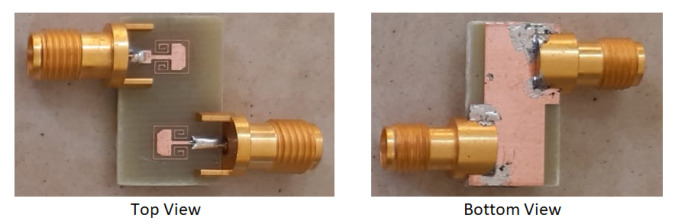
Fabricated prototype of the proposed antenna.

In this section, the theoretical observations and simulation results are compared and validated. A prototype of one of the two configurations has been developed and tested using a vector network analyzer and radiation pattern measurement setup. The realized gain of the antenna is measured using the two-antenna method. The front and back views of the prototype are shown in Figure 13. The MIMO configuration 2 is unconventional; thus, it is chosen for prototype development and testing.

The antenna is fabricated on a low-cost flame-redundant-4 (FR4) substrate. The substrate parameters, loss tangent, permittivity, and height are 0.02, 4.3, and 1.6 mm, respectively. It is well known that FR4 has higher dielectric loss in the FR2 band. This substrate is chosen to reduce the cost of experimentation. For the practical implementation, a low-loss substrate such as Rogers RT/Duroid 5880 substrate of loss tangent 0.004 is recommended. However, the change in the substrate will affect the radiation performance and slightly alter the overall size in this frequency range. If a lower-loss and lower-permittivity substrates are chosen, the antenna gain and efficiency will improve, but the size will slightly increase, and vice versa. The functioning of the proposed technique will remain the same. The printed circuit board has copper thickness of 1 oz (34.79 μm), so that the thin spiral-inspired parasitic can be properly printed. Two Johnson 145-0701-841 end-launch jacks are used to connect the antenna ports with testing equipment.

**Figure 14:**
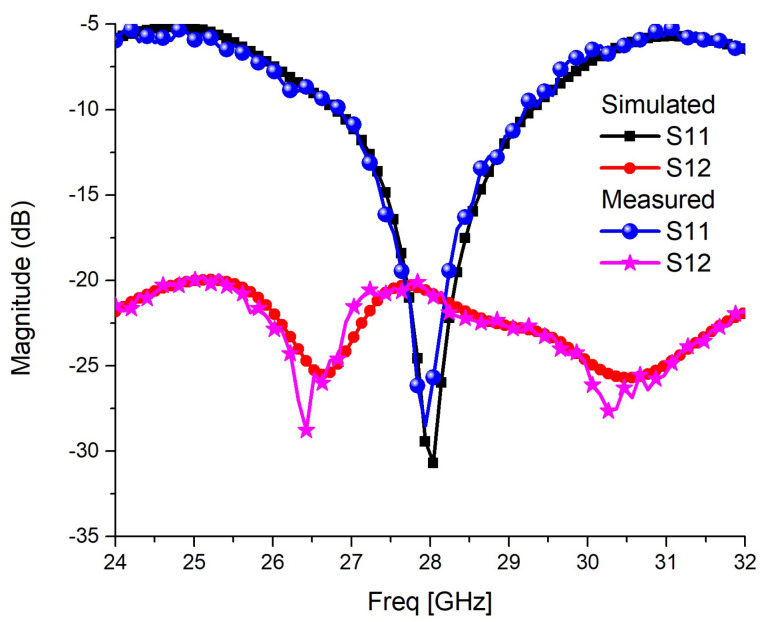
Simulated and measured scattering parameters of MIMO configuration-2.

**Figure 15:**
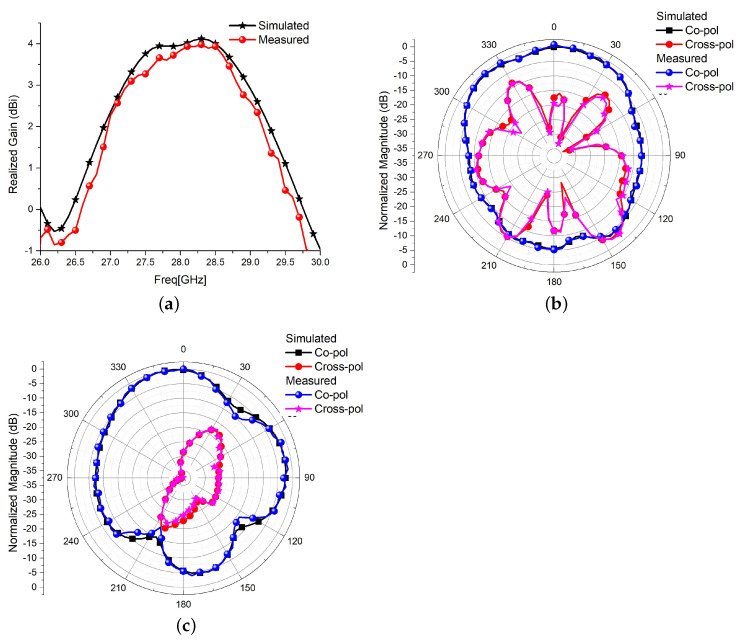
Measured results (**a**) Gain (**b**) Broadside E-plane radiation at 28 GHz (**c**) Broadside H-plane radiation at 28 GHz.

The simulated and measured impedance-matching parameter, *S*_11_, and mutual coupling parameter, *S*_12_, are shown in Figure 14. Simulated and measured scattering parameters have excellent matching, and the measured bandwidth is 8%. In the simulation model, the antenna is fed through a 50 Ω co-axial feed of core diameter 0.695 mm, substrate diameter 1.6 mm, and dielectric constant 1. An equivalent sub-miniature version-A 50 Ω connecter is used in the fabricated prototype, leading to excellent matching. The measured *S*_12_, well below −20 dB, matches with the simulated one and justifies the theoretical discussions and simulation results. The simulated and measured realized gain of the MIMO configuration 2 is shown in Figure 15a. The measured gain of the antenna nearly matches the simulated results, achieving a peak of 3.95 dBi at the centre frequency of 28 GHz. The broadside E and H-field simulated and measured radiation patterns are shown in Figure 15b,c. There is good agreement between simulation and measured radiation patterns. The E-plane cross-polarization is well below −12 dB in the main lobe of 52.5${}^{{}^{\circ}}$. The good agreement between simulation and measured antenna characteristics validates the proposed edge trimming and the loading of the spiral-inspired parasitic for the cross-polarization reduction, and isolation improvements in MIMO antennas. The proposed work is compared with some existing literature and presented in Table 3. A Figure of Merit (FoM) in terms of the ratio of two-element MIMO antenna length versus isolation in dB is also calculated and presented in Table 3. It is desired that a MIMO antenna should have a smaller size and higher isolation. Thus, the FoM should be as low as possible. It is clear in Table 3 that the proposed antenna has better FoM in terms of size and isolation. The proposed work has a larger size, but excellent isolation, bandwidth, reasonable gain, *ECC*, and *DG*. The work presented in [8] has superior simulated results, but the experimental results are not presented.

**Figure 16:**
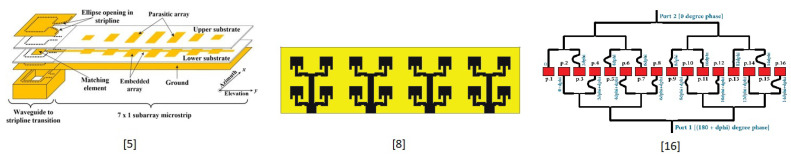
Architecture of some work reported in literature [5], [8], and [16] respectively.

To compare the architectural difference of the proposed design, the architecture of some reference designs is presented in Figure 16. In [5], a stacked structure is used, which requires some additional effort for fabrication. In [8], isolation improvement techniques are not implemented. In [16], an inverted configuration of a large single-element array is placed to form a two-element MIMO without an isolation improvement technique. In the proposed work, the inverted configuration of a trimmed-edge rectangular patch antenna with a self-isolating parasitic is presented. The obtained results are competitive with the existing design and confirm its suitability for practical applications.

## 4. Conclusions

A corner-trimmed and spiral-inspired parasitic-loaded single-element antenna is developed for 5G FR2 band MIMO applications. The forward corner trimming of a rectangular patch antenna is found to be highly effective at suppressing the cross-polarization radiation. Two MIMO antenna configurations have been developed using the proposed single element. In the linear MIMO configuration, the embedded parasitics are out of phase with the mutually exciting currents, strongly opposing the mutual coupling. In the inverted arrangement of the single element, the loaded parasitics come into phase with the mutually exciting current, and thus have lower isolation. When the antenna elements are placed in the far field, they have naturally good isolation, but overall, the antenna size becomes large. For the low-cost consumer application, the FR2 band antenna may be placed half-wavelengths apart to realize better isolation, as the wavelength is very short. The agreement between simulation and measured results validates the proposed work.

## Figures and Tables

**Table 1:** Performance of the MIMO configuration-I antenna at 28 GHz.

*d* (mm)	2	4	5	6
$S_{11}$ (dB)	−40.00	−27.77	−44.77	−26.44
BW (GHz)	2.46	3.1	2.89	2.16
Gain (dB)	4.8	5.5	5.2	4.7
Isolation (dB)	<−15	<−15	<−20	<−20

**Table 2:** Performance comparison of MIMO-1 and MIMO-2 for $d=0.54\lambda_{0}$, 28 GHz.

Parameter	MIMO-1	MIMO-2
S11 (dB)	−27.90	−30.06
BW (GHz)	2.16	3.00
Gain (dB)	4.8	4.1
Isolation (dB)	<−25	<−20
ECC (Abs)	0.01	0.03
DG (dB)	10	9.985

**Table 3:** Comparison of the proposed work with some existing FR2 band antennas in literature.

Ref./Metric	[5]	[6]	[8]	[9]	[10]	[16]	This Work
Size (mm${}^{2}$)	74.5 × 89.55	8.06 × 6.96	17.2 × 62	10 × 20	19.9 × 30	17.45 × 99.2	10.8 × 19.64
Substrate	NA	FR4	RTD 5880	Polyimide	Taconic TLY-5	RTD 5880	FR4
BW (%)	6.3	8.5	12.6	9.4	5.4	24.4	8
Gain (dB)	21.8	5.7	19.6	3.8	7.41	19.88	4.8
Isolation (dB)	13.46	NA	>30	NA	NA	20	25
*ECC*, *DG* (dB)	NA	NA	0.01, 10	NA	NA	NA	0.01, 9.99
FoM	6.65	NA	1.03	NA	NA	4.96	0.79

## Data Availability

Not applicable.
